# Efficacy of an Emotion Regulation Training in Enhancing Resilience Among Patients With Congestive Heart Failure: A Randomized Clinical Trial

**DOI:** 10.1002/hsr2.70421

**Published:** 2025-03-23

**Authors:** Fatemeh Kalij, Mohammad Akbari, Mousa Alavi, Vajihe Atashi

**Affiliations:** ^1^ Nursing & Midwifery Care Research Center Isfahan University of Medical Sciences Isfahan Iran

**Keywords:** congestive heart failure, emotion regulation, resilience

## Abstract

**Background and Aims:**

Congestive heart failure is often associated with diminished resilience in patients, leading to adverse mental health outcomes. This study aimed to explore the impact of an emotion regulation program on the resilience of individuals coping with congestive heart failure.

**Methods:**

This randomized clinical trial involved 70 hospitalized patients with congestive heart failure at Shahid Chamran Medical Center in Isfahan. Participants were randomly assigned to either the intervention group (*n* = 35) or the control group (*n* = 35). The intervention group underwent a six‐session emotion regulation program, with each session lasting 45–60 min. Data collection involved the Connor–Davidson Resilience Scale, administered at three stages: preintervention, immediately postintervention, and 1 month postintervention. Data were analyzed using SPSS version 22, employing chi‐square tests, Fisher's exact tests, *t*‐tests, and repeated measures ANOVA (significance level: *p* < 0.05).

**Results:**

The mean resilience scores for the intervention group were 37.33 ± 17.25 preintervention, 92.17 ± 3.88 immediately postintervention, and 87.26 ± 3.33 1 month postintervention, indicating a statistically significant difference (*p* < 0.001). Conversely, the control group's mean resilience scores during the same periods were 37.77 ± 25.58, 19.34 ± 13.22, and 31.80 ± 19.98, showing nonsignificant differences (*p* > 0.05). Additionally, comparisons of mean resilience scores between the intervention and control groups immediately and 1 month postintervention revealed significant differences (*p* < 0.05).

**Conclusion:**

The findings underscore the effectiveness of the emotion regulation program in enhancing resilience among patients with congestive heart failure. It is recommended that healthcare professionals, particularly nurses, incorporate this intervention into their patient care practices to foster resilience in individuals managing congestive heart failure.

## 1 Introduction

Cardiovascular diseases represent a prevalent and debilitating health challenge, contributing significantly to global mortality rates [1]. Among these, congestive heart failure (CHF) is an increasing concern within the healthcare sector [2, 3]. In addition to its grave prognosis, CHF results in physical activity limitations, social isolation, psychological distress, reduced functionality, increased dependency, anxiety regarding mortality, and a diminished quality of life [4, 5, 6]. Nearly half of individuals coping with heart failure experience troubling psychological symptoms, including guilt, despondency, low self‐esteem, fatigue, and depression [7].

The psychological challenges faced by CHF patients are further exacerbated by their low resilience. Resilience refers to the capacity for optimal adjustment in the face of adversity, threats, and stress [8]. Research has indicated a pervasive lack of resilience among patients with CHF [9, 10]. Consequently, interventions aimed at enhancing resilience in this population are imperative. Resilience is intertwined with individual attributes such as a sense of coherence, optimism, positive emotions, self‐esteem, self‐efficacy, cognitive flexibility, coping strategies, and social support, highlighting the need for interventions that bolster these critical traits [11].

In this context, emotion regulation emerges as a pivotal intervention. Emotion regulation encompasses the ability to modify or adapt one's emotional responses, representing a set of motivational processes through which individuals recognize and manage their emotions [12].

It refers to the efforts individuals make to influence the type, timing, and expression of their emotional experiences, as well as changes in the duration or intensity of emotional, behavioral, and physiological processes either automatically or intentionally, consciously or unconsciously through strategies such as reappraisal, rumination, self‐disclosure, avoidance, and restraint [13, 14, 15, 16].

**Figure 1:**
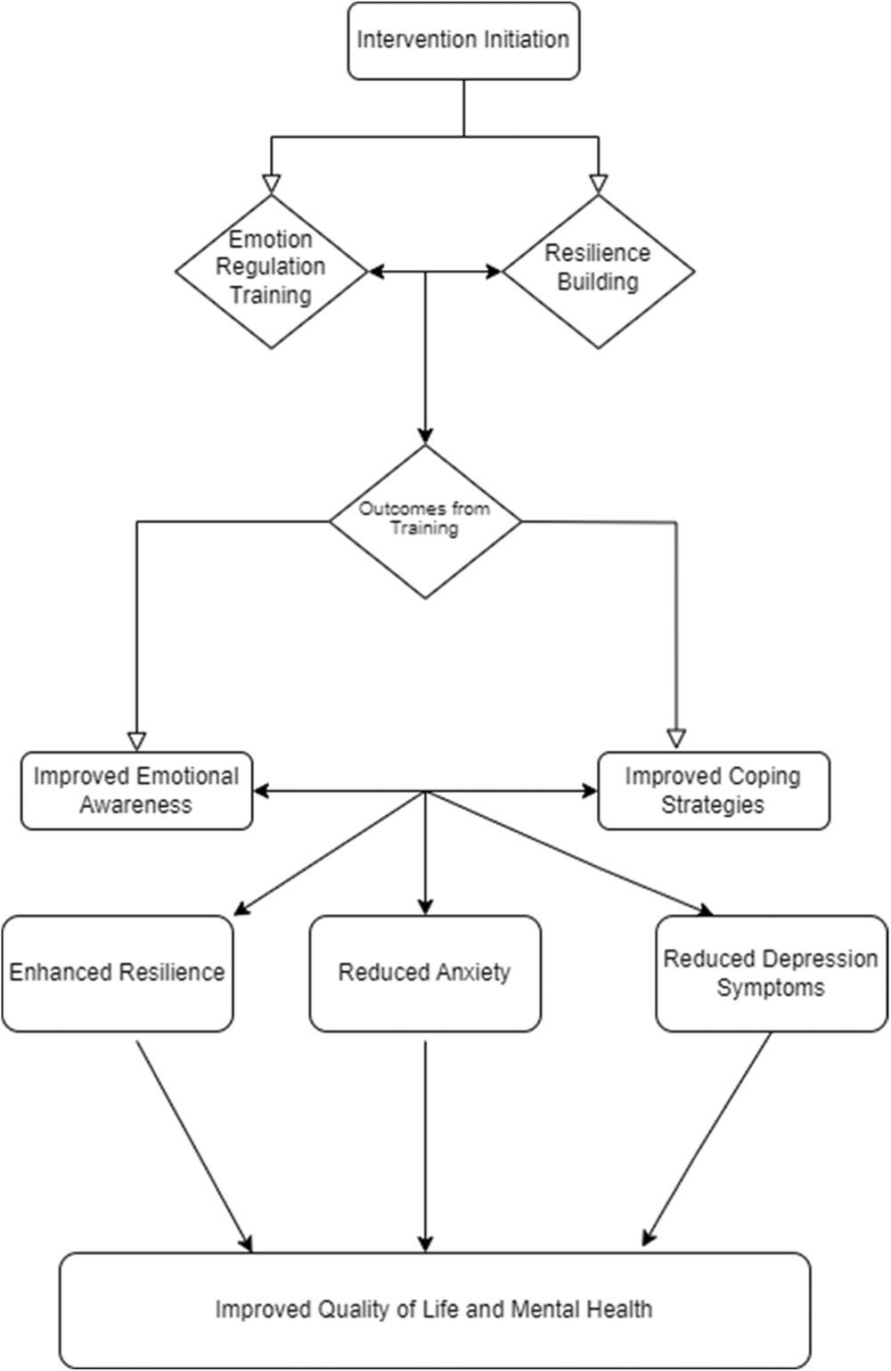
Hypothesized framework of intervention effect.

Investigations have shown that one of the emerging educational approaches, specifically emotion regulation training based on the Gross model, effectively addresses a broad spectrum of patient challenges [17, 18, 19]. The model proposed by Gross (2002) provides a conceptual framework for understanding the emotion regulation process, rooted in the quality model of emotional experience. According to Gross, each phase of the emotional experience presents potential regulatory objectives, and skills for emotion regulation can be applied at various stages within this process [11] (Figure 1).

Research outcomes have documented the positive impact of emotion regulation interventions on diverse health outcomes, including improvements in quality of life among patients with diabetes [20], reductions in worry and psychological distress in women experiencing depression [21], and enhancements in the quality of life of individuals with heart diseases [22, 23]. However, limited research has specifically explored the effects of emotion regulation programs on patients with CHF, and prior studies underscore the urgent need for psychological interventions tailored to heart patients, particularly those with CHF [24] To date, no research has examined the effects of emotion regulation programs on the resilience of patients contending with CHF.

Given the chronic and insidious nature of heart failure, it is essential to focus on the emotional regulation of these patients alongside addressing their physical challenges. Furthermore, fostering the emotional well‐being of patients plays a crucial role in enhancing their quality of life and promoting self‐care. Notably, nurses, during patient hospitalization, hold the vital responsibility of addressing this fundamental aspect of care. Thus, the current study aims to elucidate the impact of an emotion regulation program on the resilience of patients living with CHF who are receiving care at the Shahid Chamran Medical Training Center in Isfahan in 2023.

## 2 Materials and Methods

### 2.1 Study Design and Setting

The present study constituted a clinical trial conducted from January 2023 to May 2023. The research cohort comprised patients diagnosed with CHF admitted to Shahid Chamran Hospital in Isfahan, Iran.

### 2.2 Study Participants and Sampling

The sample size was determined to be 68 individuals based on the study by Zeighami Mohammadi and Hashemi [25], using the following formula with a 95% confidence level and 80% power:

$$\frac{{\left(Z_{1-\frac{\alpha }{2}}+Z_{1-\beta }\right)}^{2}(S_{1}^{2}+S_{2}^{2})}{{\left(d\right)}^{2}}.$$

**Figure 2:**
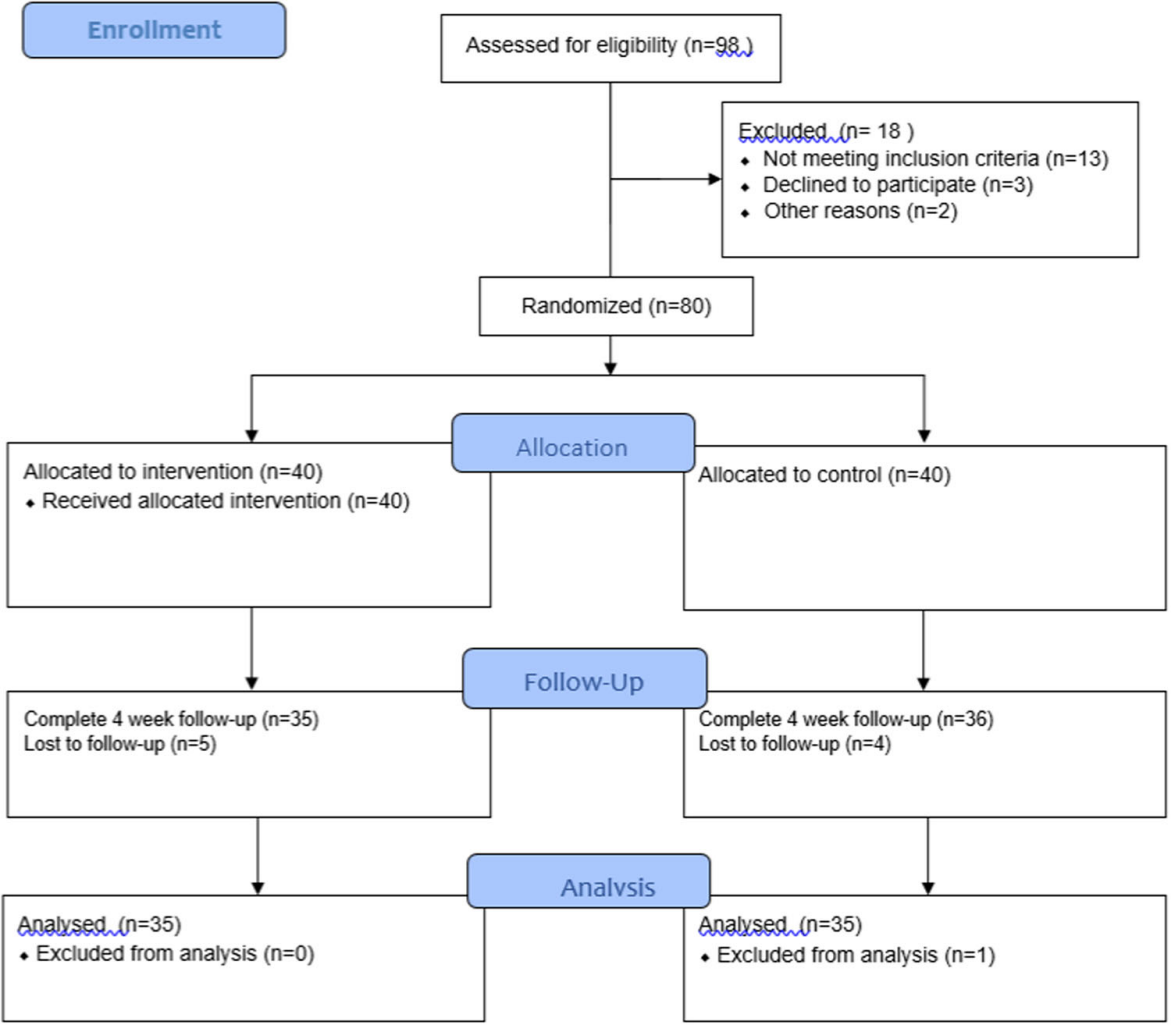
Study follow diagram.

Taking into account the possibility of sample attrition, a sample size of 75 individuals was considered. Participants were selected through convenient sampling from among patients visiting the inpatient departments of Chamran Hospital who met the study's inclusion criteria. Subsequently, participants were randomly assigned to the intervention and control groups. Random allocation was performed using a random number table. Initially, 98 patients were identified for potential inclusion, of whom 18 were excluded due to failure to meet the study's criteria or a lack of willingness to participate. Consequently, the remaining 80 individuals were randomly assigned to two distinct groups, each consisting of 40 participants. Throughout the trial, five individuals from each group were excluded due to disease exacerbation or incomplete questionnaire responses. Ultimately, the analysis encompassed data from 35 participants in each group (Figure 2).

The study's inclusion criteria encompassed patients aged 35–70 years, presenting with CHF for a minimum of 1 year, and classified as NYHA class III or IV. Additionally, patients were required to be fluent in Persian, capable of reading, writing, and comprehending the language, have no participation in emotional regulation training courses, and experience no severe stress‐inducing events, such as divorce or the loss of loved ones, within the preceding 6 months. Furthermore, the absence of mental retardation or a history of psychiatric illness requiring drug treatment, along with explicit consent to engage in the study, were essential prerequisites. Conversely, unwillingness to cooperate, the emergence of significant physical or mental issues during the study, exposure to severe stress, or nonattendance at more than two training sessions were determinant factors for exclusion.

### 2.3 Data Collection and Tools

Data collection involved the utilization of a demographic characteristics questionnaire encompassing details such as age, gender, marital status, employment status, education level, income, type of residence, number of hospitalizations, and type of home caregiver. Furthermore, the Connor–Davidson Resilience Scale (CD‐RISC), developed and psychometrically evaluated by Cannon and Davidson in 2003, was employed. This scale comprises 25 items and utilizes a 5‐point Likert scale (0 = *completely false*, 1 = *rarely true*, 2 = *sometimes true*, 3 = *often true*, 4 = *always true*) to assess resilience, with scores ranging from 0 to 100. Notably, higher scores indicate greater resilience. The reliability of the CD‐RISC was substantiated by previous studies and reports, with Cannon and Davidson verifying its reliability through the test‐retest method, yielding a Cronbach's *α* coefficient of 0.89 [26]. Similarly, Cronbach's *α* is reported to range from 0.84 to 0.93 in various studies [27].

Faculty members in the psychiatric nursing department at the Isfahan College of Nursing and Midwifery, specializing in psychology, nursing, and cardiovascular health, conducted a thorough review of the intervention materials. Their expertise ensured that the content was relevant and appropriate for the target population of CHF patients. Each faculty member assessed the materials for clarity, relevance, and applicability to the emotional regulation needs of individuals with CHF. They provided feedback on the alignment of the content with current best practices in emotion regulation and improving resilience and designed a six‐session intervention based on the Gross emotion regulation model (Table 1).

**Table 1:** Content of emotion regulation sessions based on the Gross model.

Session	Content
1	Introduction and acquaintance with the program and researcher, stating the main and secondary goals of the group, logic and stages of intervention, and framework and rules for group participation, familiarity with emotion regulation and conducting a pretest Assignment: Identifying personal goals for participating in the group
2	Understanding emotions and provocative situations through teaching various types of emotions and the short‐term and long‐term effects of emotions, the importance of learning emotion regulation skills in life Assignment: Reporting on expressing different emotions in provocative situations Identifying types of emotions in daily life
3	Reviewing previous session assignments, evaluating the vulnerability and emotional skills of members Organizing member behavior, creating change in provocative emotional situations, and teaching interpersonal communication skills Assignment: Sharing personal experiences and examples of real‐life experiences
4	Reviewing previous session assignments, expressing existence, preventing social isolation and conflict resolution, changing attention, stopping rumination and worry Assignment: Discussion, conflict resolution, reporting on changes in attention in the group
5	Reviewing previous session assignments, changing cognitive evaluations and identifying incorrect evaluations and their effects on emotional states, changing behavioral and physiological effects of emotions, confrontation, modifying behavior through changing environmental reinforcers Assignment: Implementing cognitive evaluation exercises in daily life, practicing emotion regulation tools based on using emotion regulation strategies
6	Reviewing previous session assignments, re‐evaluating and removing application barriers, summarizing topics discussed in previous sessions, creating new structures at the cognitive, behavioral, and emotional levels of individuals, conducting a posttest, appreciation, and presenting gifts to participants

The intervention was delivered by a psychiatric nurse, selected based on her relevant educational background and experience in mental health nursing. The psychiatric nurse underwent specific training to deliver the emotion regulation program, which included familiarization with the program's content, understanding the principles of emotion regulation based on Gross's model, and practicing facilitation skills. Additionally, supervision and guidance were provided by experienced faculty members to ensure adherence to the study protocol.

In the intervention group, a six‐session emotion regulation program following Gross's emotion regulation model [28] was implemented. Each session lasted 45–60 min and was conducted in groups of four to seven individuals within the ward classroom, encompassing lectures, group discussions, and a question‐and‐answer format. All participants received comprehensive training on the intervention protocol and researcher was ensured they understood the objectives and techniques to be used in the emotion regulation program.

A detailed manual outlining the procedures, session content, and strategies was provided to all of them. This manual served as a guide to maintain consistency across all intervention sessions. Participants were encouraged to provide feedback on the intervention, which helped assess engagement and satisfaction and allowed for adjustments if necessary.

Participants received guidance during the sessions on how to complete their assignments. researcher provided examples and engaged them in discussions to clarify any questions or uncertainties. Participants were asked to submit their completed assignments during the follow‐up sessions. This allowed researcher to monitor engagement and address any challenges participants faced. Participants received feedback on their assignments, which encouraged continued engagement and facilitated the application of learned skills in real‐life situations.

The control group received standard care exclusively. In cases where patients were discharged before completing the intervention (i.e., before attending all six sessions) materials were delivered virtually including instructions conducted via Skype platform.

The resilience questionnaire was completed by both groups of patients before, immediately after, and 1 month following the intervention. As a measure of research ethics, educational pamphlets and session summaries were provided to the control group upon study completion.

### 2.4 Data Analysis

Data analysis was conducted using SPSS version 22, employing statistical tests including Kolmogorov–Smirnov to evaluate data normality across both groups, Shapiro–Wilk, Fisher's exact test, chi‐square test, independent *t*‐tests, paired *t*‐tests, and ANOVA with repeated measures to compare resilience over time. One‐sided tests with a significance level of *p* < 0.05 were applied.

“All authors have read and approved the final version of the manuscript. Mohammad Akbari had full access to all the data in this study and takes complete responsibility for the integrity of the data and the accuracy of the data analysis.”

### 2.5 Ethical Considerations

This research, derived from a nursing master's thesis, was approved by Isfahan University of Medical Sciences and Services with ethics identification IR.MUI.NUREMA.REC.1401.156. All patients provided written informed consent, ensuring data confidentiality. Furthermore, the present study received approval from the Clinical Trial Registration Center under the IRCT code 20180601039934N3.

## 3 Results

This investigation involved the evaluation of 70 patients diagnosed with CHF, with 35 allocated to the intervention group and 35 to the control group (Figure 1). The mean age and standard deviation were 54.86 ± 11.70 and 59.00 ± 9.07 years for the intervention and control groups, respectively, indicating no statistically significant difference in demographic characteristics between the two groups (*p* = 0.113) (Table 2).

**Table 2:** Comparison of demographic characteristics in groups.

The investigated variable				The results of the between groups comparison: *p*‐value
		Intervention group Mean ± standard deviation	Control group Mean ± standard deviation	
Age of patients (years)		59 ± 9.07	54.86 ± 11.70	0.013*
		85.14 ± 74.82	74.14 ± 70.37	0.612*

Duration of the disease (months)	Number (Percent)	Number (Percent)	The results of the between groups comparison: *p*‐value
Gender			
Female	10 (28.57)	8 (22.85)	0.785**
Man	25 (71.42)	27 (77.14)	
Marital status			
Married	33 (94.28)	35 (52.2)	0.346***
Single	1 (2.85)	—	
Divorced	1 (2.85)	—	
Job			
Employed	9 (25.71)	8 (22.85)	0.604***
Unemployed	10 (28.57)	6 (17.14)	
Retired	9 (25.71)	13 (37.14)	
Housewife	7 (20)	8 (22.85)	
Level of education			
High school	20 (57.14)	25 (71.42)	0.225***
Diploma	13 (37.14)	9 (25.71)	
University education	2 (5.71)	1 (2.85)	
Income status			
Less than living expenses	32 (94.28)	29 (82.85)	0.247***
Equal to living expenses	2 (5.71)	5 (14.28)	
More than living expenses	1 (2.85)	1 (2.85)	
Number of hospitalizations			
First hospitalization	5 (14.28)	5 (14.28)	0.729***
Second hospitalization	5 (14.28)	7 (20)	
Third hospitalization	10 (28.57)	8 (22.85)	
Hospitalized more than three times	15 (42.85)	15 (42.85)	
Caregiver			
Spouse	20 (57.15)	18 (51.43)	0.390***
Child	10 (28.57)	11 (31.43)	
Brother or sister	2 (5.71)	3 (8.57)	
Parents	1 (2.85)	2 (5.71)	
Others	2 (5.71)	1 (2.85)	
Where they live			
City	30 (85.71)	23 (65.71)	0.093**
Village	5 (14.28)	12 (34.28)	

* Independent *t*‐test.

** Chi‐square test.

*** Fisher's exact test.

Normality testing using the Shapiro–Wilk test for the resilience variable across all three time periods in both intervention and control groups yielded nonsignificant results (*p* > 0.05), signifying normality of the research variable. The initial comparison of mean resilience scores between the intervention (37.33 ± 17.25) and control (37.77 ± 25.58) groups before the intervention revealed no statistically significant difference (*p* > 0.05). However, significant disparities emerged postintervention, with the intervention group showcasing substantial increases, resulting in mean scores of 92.17 ± 3.88 immediately after the intervention and 87.26 ± 3.33 1 month later. Conversely, the control group's mean scores immediately and 1 month after the intervention were 19.34 ± 13.00 and 31.80 ± 19.98, respectively, demonstrating significant differences between the intervention and control groups (*p* < 0.05). Utilizing a single‐variable repeated measures analysis of variance to verify the sphericity hypothesis, Mauchly's test (*p* = 0.005) indicated a rejection of the sphericity hypothesis, prompting the use of the Greenhouse–Geisser test. Overall, the within‐subjects effect was deemed significant, as evidenced by the interaction effect of time × group and time (*p* < 0.001). Further scrutiny through repeated measures analysis of variance confirmed a noteworthy distinction in mean resilience scores among patients with CHF before, immediately after, and 1 month postintervention in both the intervention group (*p* < 0.05) and the control group (*p* < 0.05) (Table 3).

**Table 3:** Comparison of the mean resilience score in patients with CHF before, immediately, and 1 month after the intervention across two groups.

Variable	Group	Before the intervention		Immediately after the intervention		One month after the intervention		Repeated measures ANOVA		
		Mean	Standard deviation	Mean	Standard deviation	Mean	Standard deviation	Test statistics	Degree of freedom	*p*‐value
Resilience	Intervention	34.37	17.25	92.17	3.88	87.26	3.33	167.86	2	< 0.001
	Control	37.77	25.58	19.34	13.22	31.80	19.98	7.72	2	0.002

## 4 Discussion

The primary objective of this study was to evaluate the impact of the emotion regulation program on resilience among patients with CHF, revealing a notable increase in the mean resilience score within the intervention group over time. Consistent with the current findings, outcomes from Ramazani et al. [29] and Pasandideh and Zare [30] demonstrated the efficacy of emotional regulation techniques in enhancing resilience levels among patients with coronary heart disease over a 1‐month period. Additionally, a study by Kim and Lee [31] indicated that cognitive emotional regulation strategies play a mediating role between stress responses and resilience levels, thereby helping to enhance resilience in patients by reducing stress. These findings align with those of the present study.

Furthermore, outcomes from Cai et al. [32] demonstrated similar efficacy of emotional regulation techniques in augmenting resilience levels among Chinese soldiers. Luque et al. elaborated on the impact of emotional regulation interventions in individuals with cardiovascular diseases, emphasizing the effectiveness of such interventions in promoting emotional balance, enhancing stress and anxiety regulation skills, and bolstering self‐efficacy in managing negative emotions during stressful situations. Their intervention led to a significant increase in resilience levels during the month following the intervention [33].

The parallelism of these findings with the current study underscores the potential of the emotional regulation program to yield analogous outcomes for the patients in this investigation. Moreover, the concurrence between the findings of the aforementioned studies and the current investigation highlights that the strategies employed in cognitive emotional regulation interventions resemble a strategic approach aimed at enhancing positive refocusing, fostering an optimistic outlook, and improving problem‐solving capabilities to navigate the emotional impacts of stressful situations, such as heart disease and its associated complications [29].

The results of studies by Mehboodi, Amiri, and Molavi [34] and González‐Flores et al. [35] indicated that emotion regulation interventions can increase the resilience levels of patients with chronic kidney failure up to 1 month after the intervention [34, 35], which is consistent with the findings of the present study. Although there are differences in the type of disease between these studies and the current study, the chronic nature of all three conditions suggests that emotion regulation aids in understanding, modifying, and experiencing emotions in patients with chronic diseases. This enhances their ability to cope with stressful situations, such as chronic kidney disease and CHF, by improving their capacity to tolerate negative emotions and experience more positive emotions, potentially leading to better adaptation to the challenges of disease treatment and enhanced resilience [34].

Moreover, findings by Baziliansky and Cohen [36] align with those of the present study, highlighting the transformative impact of emotion regulation interventions on elevating resilience levels in patients contending with colorectal cancer, particularly over an 8‐week intervention period.

The results from Ramazani et al. [29] confirmed the effectiveness of the emotional regulation method based on the Gross model in improving resilience levels among patients with coronary heart disease during a 1‐month intervention, further supporting the findings of the present study.

These results suggest the viability of employing emotion regulation interventions in the context of chronic diseases such as cancer or CHF, characterized by taxing treatment regimens and disease‐related complications. Such interventions may mitigate negative emotional responses, leading to reduced psychological distress and an enhanced cognitive and emotional profile through the identification and management of automatic and ineffective thoughts [37].

By cultivating emotional awareness, conflict resolution, and stress management, patients may enhance their capacity to tolerate and manage challenging disease conditions. This process can potentially augment problem‐solving abilities and resilience, consistent with the dynamics observed in the present study [34]. In general, emotional regulation appears to play a pivotal role in diminishing negative emotional responses while concurrently bolstering resilience in challenging circumstances. It empowers individuals to identify and manage automatic thoughts, thereby mitigating stress and enhancing resilience. Despite an extensive search, no contradictory studies were identified.

## 5 Limitations and Strengths

Due to reliance on questionnaire‐based assessments, potential variations in resilience interpretation or biases in questionnaire completion may have impacted the study's outcomes. Future investigations could benefit from qualitative methodologies and interviews to delve deeper into the impact of emotion regulation programs on resilience among CHF patients. Moreover, the physical state (e.g., severity of dyspnea, weakness, and lethargy) and mental conditions (e.g., severity of anxiety and stress) of participants may have influenced their responses, representing potential limitations.

On the positive side, this study is the first of its kind in Iran, exploring the effects of an emotion regulation program on resilience among NYHA class III and IV heart disease patients. This pioneering research can provide a foundation for future endeavors in similar domains.

## 6 Conclusion

The study's findings underscore the significant enhancement of resilience in CHF patients due to the emotion regulation program. Consequently, it is recommended that nurses receive specialized training in this area to augment the resilience of heart failure patients. This calls for strategic managerial planning to empower nurses in designing and implementing training programs that incorporate emotion regulation promotion. In doing so, nurses can fortify the caregiving system, expand their roles in patient care, and contribute to the development of resilience among CHF patients.

## 7 Application of Research Findings

The research on emotion regulation programs for patients with CHF has implications across clinical, educational, management, and research domains.

*Clinical Field:* Nurses can improve resilience in patients with CHF by implementing an emotion regulation program. This program can increase emotional self‐efficacy, resilience, and compliance with medication regimens.

*Education Field:* Nurses should be trained to provide emotional regulation programs to patients with heart failure. This training should be included in nursing curricula, and workshops and in‐service training can further help nurses develop this skill.

*Research Area:* The findings of this study can lead to further research on emotion regulation programs for patients with CHF. Future studies can explore the impact of these programs on mental health and treatment adherence.

*Management Area:* The emotion regulation program represents a low‐cost, effective method to improve emotional self‐efficacy, resilience, and medication adherence in patients with CHF. It should be implemented as a necessary training course for cardiac nurses.

## 8 Suggestions for Future Research

The findings of this study can pave the way for researchers to explore the effects of emotion regulation programs on emotional self‐efficacy, resilience, and medication adherence among patients with CHF. Based on the results of this study and the importance of mental health and adherence to drug treatment in heart failure patients, the following items are suggested for further research:

– Investigate the relationship between emotional self‐efficacy, resilience, and treatment adherence in heart failure patients.
– Conduct qualitative studies to explore the experiences of heart failure patients regarding emotion regulation in managing their condition.

## Author Contributions

**Fatemeh Kalij:** conceptualization, data curation, formal analysis, writing – review and editing, writing – original draft. **Mohammad Akbari:** methodology, validation, visualization, writing – review and editing, software, formal analysis, project administration, supervision, resources. **Mousa Alavi:** methodology, visualization, project administration, formal analysis, software, supervision. **Vajihe Atashi:** validation, formal analysis.

## Conflicts of Interest

The authors declare no conflicts of interest.

## Transparency Statement

The lead author Mohammad Akbari affirms that this manuscript is an honest, accurate, and transparent account of the study being reported; that no important aspects of the study have been omitted; and that any discrepancies from the study as planned (and, if relevant, registered) have been explained.

## Data Availability

The data that support the findings of this study are available from the corresponding author upon reasonable request. The data sets generated and/or analyzed during the current study are not publicly available to protect the confidentiality of the participants.
